# The Effect of Waste Low-Density Polyethylene/Plasticizer Diisononyl Phthalate on the Performance of Asphalt Binder

**DOI:** 10.3390/ma18112580

**Published:** 2025-05-31

**Authors:** Peng Hu, Xiao Shao, Kun Wang, Haichuan Jia, Long Chen

**Affiliations:** School of Transportation and Civil Engineering, Shandong Jiaotong University, Jinan 250357, China; 204021@sdjtu.edu.cn (P.H.); 22107046@stu.sdjtu.edu.cn (X.S.); jiahaichuan@sdjtu.edu.cn (H.J.); 204151@sdjtu.edu.cn (L.C.)

**Keywords:** modified asphalt, waste LDPE, plasticizer DINP, rheological properties

## Abstract

As an aspect of green road construction, the use of waste plastic agricultural film in asphalt pavement not only mitigates environmental pollution but also enhances the mechanical properties of asphalt. However, it has been plagued by problems such as poor low-temperature crack resistance and poor compatibility. To address this problem, this study used waste low-density polyethylene (LDPE), sourced from waste film, and the plasticizer diisononyl phthalate (DINP) to enhance the properties of asphalt. Based on orthogonal tests, rheological property tests, conventional property tests, storage stability tests, fluorescence microscopy (FM), and Fourier transform infrared (FTIR) tests, waste LDPE/plasticizer DINP-modified asphalt samples were evaluated. Orthogonal tests indicated that a modified asphalt optimum preparation process of 30 min and 4500 rpm at 180 °C was beneficial. Conventional and rheological property tests revealed that 4% waste LDPE modified with 2.5% plasticizer DINP represents the optimal combination to effectively enhance the low-temperature rheological properties of asphalt while exerting minimal impact on its high-temperature characteristics. Storage stability and FM analysis indicated that waste LDPE is evenly dispersed in the modified asphalt binder when 3% plasticizer DINP is added. FTIR analysis revealed no change in the absorption peaks after waste LDPE and plasticizer DINP were added to asphalt, indicating that no chemical reactions occurred. Overall, waste LDPE/plasticizer DINP-modified asphalt exhibits excellent rheological properties and storage stability, which are conducive to green road construction and resource utilization.

## 1. Introduction

In recent years, the rapid development of the plastics industry has led to the accumulation of large quantities of waste plastics (used agricultural films). This has given rise to increasingly prominent environmental pollution problems and economic challenges [1,2]. Many researchers have conducted studies that used modified asphalt and added polymers to asphalt to modify it, which has resulted in improvements in the green and sustainable development of road construction [3,4,5,6,7]. The modification of virgin binders with resin-based polymers has emerged as an effective method in modified asphalt technology in recent research [8,9,10]. Waste plastics such as polyethylene (PE) have undergone significant advancements in recent years. The addition of waste plastics can not only improve the performance of asphalt pavement but also positively affect the sustainable development of the environment.

In agricultural production, PE waste films constitute the majority of commonly used waste films, and primarily comprise high-density polyethylene (HDPE) and low-density polyethylene (LDPE), while low-molecular-weight LDPE is more effective and suitable as an asphalt modifier than HDPE [11,12]. Relevant studies have pointed out that the raw material LDPE in waste plastics can effectively improve the high-temperature rutting resistance of modified asphalt [13,14,15,16]. Through investigation of the effects of LDPE on the rheological/viscoelastic properties and aging behavior of asphalt, it was found that the incorporation of waste plastics can effectively enhance the anti-rutting and fatigue properties of asphalt [17]. Birlie selected LDPE as an asphalt modifier to study the effects of temperature, the dosage, and the shear time on the properties of PE-modified asphalt. The results showed that the addition of LDPE improved the high-temperature softening point of the asphalt and affected its low-temperature ductility [18]. Ming and Dalhat studied the rheological properties and compatibility of LDPE-modified asphalt binder and reported that 4% LDPE had optimal compatibility with asphalt, but that excessively high content led to poor compatibility [19,20]. In another study, asphalt was modified using desulfurized rubber (DR) and LDPE. The results showed that the penetration, ductility, softening point, and penetration index of DR and LDPE composite-modified asphalt were 30.2%, 22.3%, 3.5%, and 11.1% higher than those of rubber crumb-modified asphalt (CRMA), respectively [21]. In addition, some researchers have combined LDPE with ethylene vinyl acetate copolymer (EVA) to modify asphalt, improving its viscoelasticity and high-temperature rheology [22,23]. In general, waste LDPE, as a modifier, has demonstrated excellent performance in terms of the high-temperature rutting resistance of asphalt, but not in terms of its low-temperature properties or compatibility. Therefore, identifying suitable additives to improve the low-temperature performance, compatibility, and overall properties of LDPE is crucial

It has been reported that the low-temperature properties and compatibility of asphalt can be improved by adding various additives [24], among which plasticizers are the most prominent in terms of their low-temperature performance [25,26]. Gao revealed the compatibility mechanism of different plasticizers and virgin binders through molecular dynamics (MD) simulation. It was found that the plasticizer Trioctyl Trimellate (TOTM) has the best stability in asphalt modification, and that the plasticizer Acetyl tributyl citrate (ATBC) has the worst [27]. Tian found that 2% DOA-modified asphalt had the best low-temperature properties among the four plasticizer-modified asphalt samples that they tested, but it was susceptible to high-temperatures [28]. Taken together, plasticizers are known to improve the low-temperature cracking resistance of asphalt, while LDPE improves the high-temperature deformation resistance of asphalt. Therefore, using plasticizers and low-density polyethylene in asphalt may provide a new approach to improving the properties of asphalt binder.

In addition, the degree of mixing of polymer-modified asphalt is critical to its performance. Inadequate mixing can greatly affect the structural instability of asphalt and lead to segregation. The previous literature on polymer-modified asphalt preparation mostly addresses direct mixing/shearing, where the polymer modifier and asphalt are directly mixed and stirred at a certain temperature for a certain amount of time to complete the preparation of modified asphalt [29,30]. However, the conditions, such as the time and temperature, of this method are mostly selected through the experience of the researchers, and there is no in-depth study on the effect of the preparation parameters on the performance of modified asphalt. In addition, the current research also details the pretreatment of modified asphalt through the direct addition of polymer modifiers into the asphalt. Xia detailed the pretreatment of a PE modifier with the help of a twin-screw extruder to prepare a high-performance PE composite modifier so that the modified asphalt formed a relatively stable network structure [31]. Similarly, in another study, plastics and rubber were heat cracked and desulfurized by a twin-screw extruder to complete the preparation of a rubber polymer composite change agent, which was then added to the asphalt to complete the preparation of modified asphalt with improved storage stability [32]. However, the pretreatment modifier method requires the addition of a catalyst to crack the polymer during the reaction process, and its temperature control is unstable. Most of the current studies have investigated modifiers in asphalt under the same blending conditions, without in-depth optimization of the effect of blending parameters on the performance of modified asphalt. Therefore, the optimal preparation process parameters of composite modified asphalt need to be investigated in subsequent studies to ensure the preparation of modified asphalt with excellent performance.

**Figure 1:**
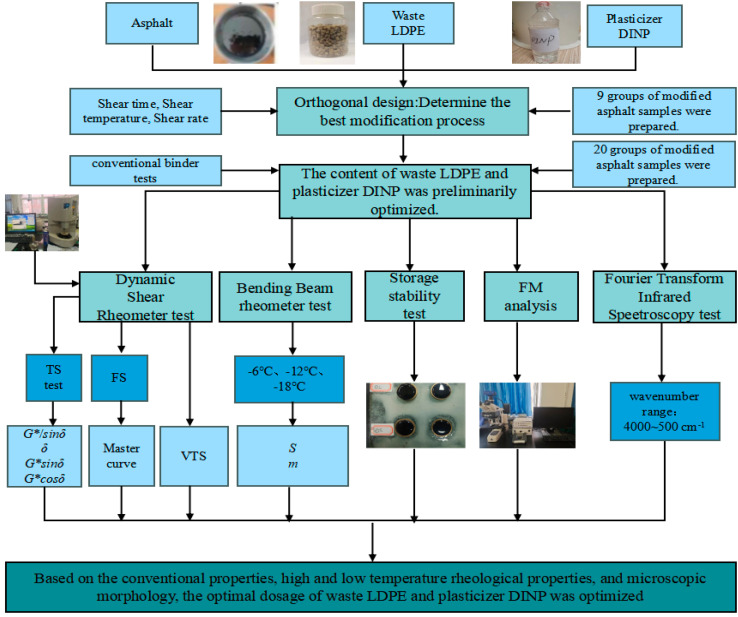
Experimental framework of this study.

This study aims to identify the optimal modification process parameters and blending amounts for waste LDPE/plasticizer DINP-modified asphalt, and to evaluate the feasibility of improving the physicochemical and rheological properties of asphalt binder via the addition of waste LDPE with plasticizer DINP. The optimum process parameters for waste LDPE/plasticizer DINP-modified asphalt binder were determined through orthogonal experiments. The rheological properties, compatibility, and microstructure of waste LDPE/plasticizer DINP-modified asphalt were evaluated via conventional property tests, dynamic shear rheometry (DSR), bending beam rheometry (BBR), segregation tests, FM tests, and FTIR spectroscopy, and the optimum dosage of waste LDPE and plasticizer DINP was finally optimized. This research has great significance for engineering practice and the promotion of waste plastic-modified asphalt. The specific process of the conducted tests is shown in Figure 1.

## 2. Materials and Sample Preparation

### 2.1. Asphalt

The PG 70-22 asphalt binder used in this study was produced by Qilu Petrochemical Company in Jinan, China, and its basic properties meet ASTM D5, D36, and D113 standards [33,34,35]. The penetration (25 °C, 5 s, 100 g) was 68.41/0.1 mm, the softening point was 46.6 °C, the ductility (10 °C) was 76.9 cm, and the ductility (15 °C) exceeded 150 cm.

### 2.2. Waste LDPE

**Figure 2:**
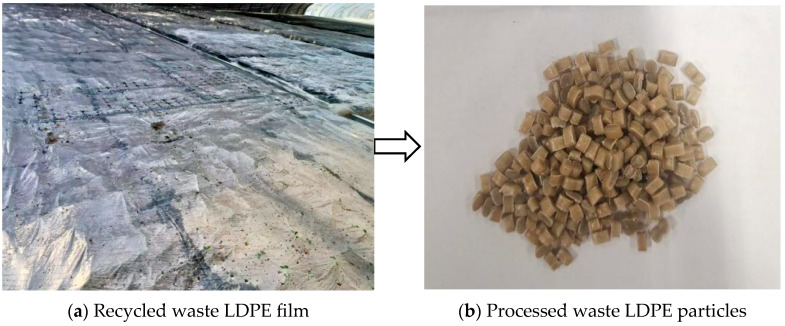
Appearance of waste LDPE before and after production and processing.

Waste LDPE was purchased from a waste recycling station in Tongcheng City, China. The main raw material was recycled waste LDPE film, and its processing flow was as follows: waste mulch screening, rinsing and drying, crushing, melt extrusion, and pelletizing to get waste LDPE pellets. The appearance of the material before and after the production process is depicted in Figure 2. The main technical indicators are shown in Table 1.

### 2.3. Plasticizer DINP

**Figure 3:**
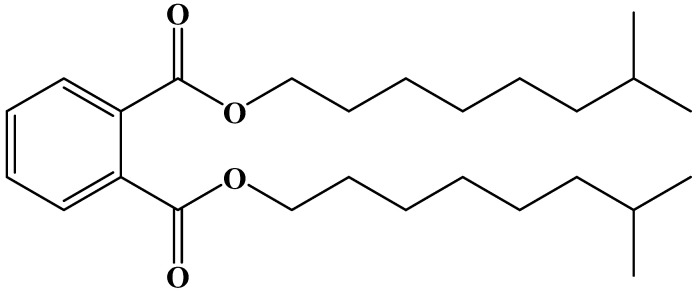
Chemical structure of the plasticizer DINP.

The plasticizer DINP is a transparent, colorless, and odorless oily liquid with the chemical formula of C_26_H_42_O, and its chemical structure is shown in Figure 3. The density was 0.971 g/cm^3^, the flash point was 210 °C, the water content was 0.1%, and the purity was 99.71%. It should be noted that, while the plasticizer DINP improves asphalt properties, relevant studies have shown that it is potentially harmful to the reproductive system and should not be used in toy manufacturing [36]. Given this, during the test, to ensure the safety of the researchers, we all strictly followed the laboratory safety operation procedures and took appropriate protective measures.

### 2.4. Sample Preparation

**Figure 4:**
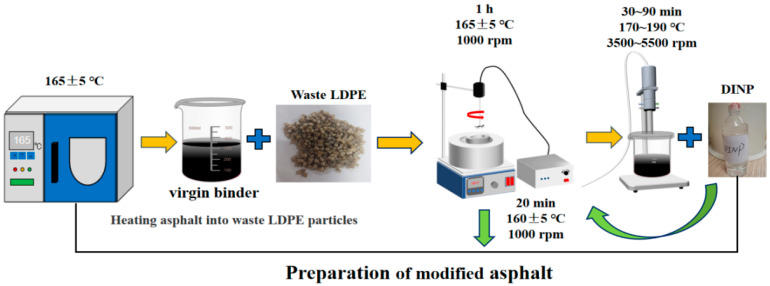
Modified asphalt preparation process.

According to the relevant literature and preliminary indoor research [37,38], waste LDPE/plasticizer DINP-modified asphalt was prepared by selecting a 4% waste LDPE and 3% plasticizer DINP ratio. The preparation process was divided into three stages. First, the virgin binder was heated to 165 ± 5 °C. Waste LDPE, with a predetermined mass ratio, was added and stirred at a constant speed of 1000 rpm for 45 min to allow for homogeneous dispersion and full expansion. The waste LDPE and virgin binder were subsequently heated to 170~190 °C, and a high-speed shearing machine was operated at 3500~5500 rpm for 30~90 min, shearing and grinding the LDPE modifier to obtain waste LDPE-modified asphalt. Finally, the temperature was reduced to 160 ± 5 °C, and the predetermined ratio of plasticizer DINP was added. The residual bubbles were removed by stirring with a mixer at 1000 rpm for 20 min to complete the preparation of waste LDPE/plasticizer DINP-modified asphalt. The preparation process is shown in Figure 4.

## 3. Test Methods

### 3.1. Orthogonal Experiment

To determine the optimal process parameters for the modification of asphalt with waste LDPE/plasticizer DINP, orthogonal experiments were conducted [39,40]. Since modified asphalt is mainly prepared by high-speed shearing, the shear process is crucial, so this study was conducted by selecting the shear rate, shear temperature, and shear time as the influencing factors, a total of 9 groups of experiments were conducted, and orthogonal tests were carried out [41]. The amount of waste LDPE and plasticizer DINP in each group was 4% and 3%, respectively. Furthermore, the optimal process parameters for asphalt modification were ultimately ascertained by assessing the fundamental properties of the nine groups of composite-modified asphalt, including the softening point, penetration at 25 °C, and ductility at 5 °C. The orthogonal experimental designs for different factors and levels is shown in Table 2.

### 3.2. Conventional Tests

To analyze the effects of the waste LDPE and plasticizer DINP dosage on the conventional properties of modified asphalt, 2%, 3%, 4%, and 5% waste LDPE and 0%, 2%, 2.5%, 3%, and 3.5% plasticizer DINP dosages were selected. The 20 groups of waste LDPE/plasticizer DINP-modified asphalt samples were prepared via the optimal modification process described above. Through the penetration test, a softening point test (global method), 5 °C ductility test, and 25 °C elastic recovery rate test were carried out to determine the physical properties of waste LDPE/plasticizer DINP-modified asphalt, and a preliminary analysis of the optimal mixing of waste LDPE/plasticizer DINP-modified asphalt was performed. The penetration and softening points were used to evaluate the high-temperature properties of asphalt, ductility was used to evaluate its low-temperature properties, and the elastic recovery rate index was used to evaluate its fatigue resistance.

### 3.3. Temperature Scanning Test

To further determine the optimal blend of waste LDPE/plasticizer DINP-modified asphalt, a temperature scanning test employing a dynamic shear rheometer (DSR) was conducted. The samples were loaded in constant strain control mode via a parallel plate with a diameter of 25 mm and a gap of 1 mm. The temperature scanning interval was set to range from 58 to 82 °C, with a temperature interval of 6 °C and an angular frequency of 10 rad/s. Finally, the prepared samples were placed to run the test. Experimentally measured complex modulus (G*), phase angle (*δ*), and calculated rheological indices (the rutting factor (G*/sin *δ*), loss modulus (G*sin *δ*), and storage modulus (G*cos *δ*)) were used to comprehensively evaluate the high-temperature rheological properties of waste LDPE/plasticizer DINP-modified asphalts in different categories.

### 3.4. Viscosity-Temperature Susceptibility Test (VTS)

The temperature sensitivity of modified asphalt is an important indicator that reflects the rheological properties of the asphalt and which visually characterizes its high and low-temperature properties and durability. The conventional PI value is susceptible to artificial and temperature factors, reducing the accuracy of the results [42]. Therefore, to accurately reflect the temperature sensitivity of asphalt, this study calculates the equivalent viscosity based on the results of the temperature scanning test and obtains the viscosity-temperature index (*VTS*) [43], which is used to evaluate the temperature sensitivity of waste LDPE/plasticizer DINP-modified asphalt. The calculation formulas for the equivalent viscosity and *VTS* are shown in Equations (1) and (2).

(1) $$\eta^{*}=\frac{G^{*}\delta^{-4.8628}}{\omega }$$

where *δ* is the phase angle, °; G* is the complex modulus, kPa; and *ω* is the angular frequency, rad/s.

(2) $$VTS=\frac{lg(lg\eta_{1}-lg\eta_{2})}{lgT_{1}-lgT_{2}}$$

where *T*_1_ and *T*_2_ are separate temperatures for different test points (in degrees Rankine), and where *η*_1_ and *η*_2_ are the viscosities at two temperature conditions, Pa∙s.

### 3.5. Frequency Sweep Test

The frequency scanning test for asphalt was conducted according to AASHTO T315 [44]. The temperature range was set between 30 °C and 70 °C, with intervals of 10 °C; the angular frequency spanned from 0.1 to 100 rad/s, and the sample diameter was 25 mm. Due to asphalt being a viscoelastic material, its mechanical properties are not solely dependent on temperature or time effects but rather on the equivalent effects of time and temperature combined. Therefore, to study the effects of waste LDPE and plasticizer DINP on the high-temperature rheological properties of asphalt, based on the time-temperature superposition principle (TTSP) and Williams–Landel–Ferry (WLF) equation, the reference temperature was selected to construct the complex modulus master curve [45]. And fit the dynamic modulus master curve with a Sigmoidal model [46]. The WLF equation and sigmoidal function are shown in Equations (3) and (4).

(3) $$\log\alpha_{T}=\frac{-C_{1}\times \left(T\;-T_{r}\right)}{C_{2}+\left(T\;-T_{r}\right)}$$

where *T* is the test temperature, *T*_r_ is the reference temperature, *α_T_* is the horizontal displacement factor of the test temperature *T* relative to the reference temperature *T_r_*, and *C*_1_ and *C*_2_ are the nonlinear fitting parameters due to the change in the temperature material.

(4) $$\log G^{*}=\delta +\frac{\alpha }{1+e^{\beta +\gamma logf_{r}}}$$

where G* is the complex modulus (kPa); *δ* is the minimum value of the modulus principal curve; *α* is the difference between the maximum and minimum values of the complex modulus principal curve; *β*, *γ* are the fitting parameters of the complex modulus principal curve; *f_r_* is the frequency shift.

### 3.6. Bending Beam Rheometer Test

The low-temperature rheological properties of the modified asphalt were evaluated via the BBR test. The stiffness modulus *S* and creep rate *m* were used as the evaluation indices for the low-temperature rheological properties of the modified asphalt. The tests were carried out at −6 °C, −12 °C, −18 °C, and −24 °C, and the stress continuous loading time was 240 s. Two parallel samples were made for each group of waste LDPE/plasticizer DINP-modified asphalt with different dosages.

### 3.7. Storage Stability Test

To evaluate the high-temperature storage stability of waste LDPE/plasticizer DINP-modified asphalt, the softening point of the optimally blended waste LDPE/plasticizer DINP-modified asphalt was determined via the ASTM method D5644 and compared with the optimally blended waste LDPE-modified asphalt without a plasticizer. After the modified asphalt was sealed with a standard aluminum tube, it was stored vertically in an oven at 163 °C for 48 h. After cooling, the standard aluminum tube was cut into three equal parts, and only the upper and lower ends were left for the softening point test. The high-temperature storage stability was evaluated according to the difference in the softening point between the upper and lower modified asphalts.

### 3.8. Fluorescence Microscopy (FM)

Fluorescence microscopy is a conventional microscopic method for evaluating the storage stability of asphalt. In this study, a drop of an asphalt sample was sandwiched between a slide and a cover slide and placed on a fluorescence microscope stage. The sample was observed at a magnification of 400,000, and the microscopic distribution of the modified asphalt was analyzed to further evaluate the storage stability of the waste LDPE/plasticizer DINP-modified asphalt.

### 3.9. Fourier Transform Infrared Spectroscopy (FTIR)

FTIR was used to analyze the changes in chemical functional groups before and after the modification of asphalt by waste LDPE and plasticizer DINP, and the modification mechanism of waste LDPE/plasticizer DINP-modified asphalt was studied. The wavenumber range was 4000~500 cm^−1^, and the resolution was 4 cm^−1^.

## 4. Results and Discussion

### 4.1. Orthogonal Test Analysis

Waste LDPE/plasticizer DINP-modified asphalt with different combinations of factors was prepared via orthogonal tests (4% waste LDPE and 3% plasticizer DINP were selected for this experiment). The results of the orthogonal tests are shown in Table 3.

To more accurately determine the optimal blending parameters for asphalt with waste LDPE/plasticizer DINP, the test results of the orthogonal tests were analyzed. As shown in Table 4, Table 5 and Table 6. The test results of the orthogonal tests were used to determine the optimum level for each influencing factor by calculating the K-value and *R*-value. *K_i_* is the sum of the test results corresponding to the same level of a factor; *k_i_* is the average of *K_i_* at the same level, and *R* is the variance (extreme variance) between levels. The greater the extreme value difference *R*, the greater the influence of this factor on the evaluation index; the formula is given in Equation (5) [47].

(5) $$R=\max(k_{i})-min(k_{i})$$

Table 4, Table 5 and Table 6 depict that, in terms of the softening point, the largest value for the variance *R* is that of the shear temperature, and the shear temperature has the greatest influence on the softening point. This factor corresponds to the highest value of *k* under the two-level condition (180 °C), demonstrating optimal high-temperature rutting resistance performance. This suggests that the optimal blending parameter is A_1_B_2_C_3_. For penetration, the largest variance *R* is that of the shear time, and the largest influencing factor for penetration is the shear time. This factor corresponds to the highest value of *k* at the one-level condition (30 min), suggesting that the optimal blending parameter is A_1_B_2_C_2_. Furthermore, Table 6 shows that the factor with the greatest influence on ductility at 5 °C is the shear rate, and the variance *R* is the largest under the shear rate factor. This factor corresponds to the highest value of *K* under the two-level condition (4500 rpm), and the optimal blending parameter is A_1_B_1_C_2_. Therefore, a comprehensive analysis revealed that the optimal modification process for preparing waste LDPE/plasticizer DINP-modified asphalt is a shear time of 30 min, a shear temperature of 180 °C, and a shear rate of 4500 rpm.

### 4.2. Conventional Physical Properties

**Figure 5:**
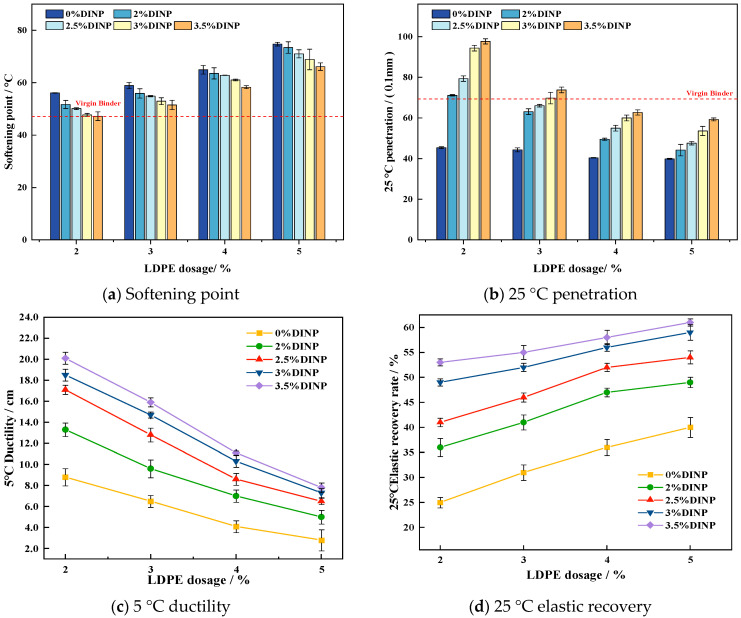
Conventional property results.

In order to analyze the effect of plasticizer DINP on the conventional properties of waste LDPE-modified asphalt, 20 groups of waste LDPE/plasticizer DINP-modified asphalt specimens were tested for their softening point, 25 °C needle penetration, 5 °C elongation, and elastic recovery, and the results of the tests are shown in Figure 5.

Figure 5a shows that, as the dosage of waste LDPE increases, the softening point tends to increase, thereby increasing the stiffness of the asphalt and improving its resistance to rutting at high temperatures. The softening points of the 4% and 5% waste LDPE-modified asphalt are as high as 60 °C and above, 31.7% and 51.6% above the softening point of the virgin binder, respectively. However, at the same waste LDPE dosage, with the addition of plasticizer DINP, the softening point began to decrease, and the high-temperature performance of the modified asphalt was affected. Among the different dosages, the softening point of modified asphalt with the addition of 2%, 2.5%, 3%, and 3.5% DINP dosages at a 2% waste LDPE dosage had the largest percentages of softening point decrease, decreasing by 7.89%, 10.69%, 14.97%, and 15.88%, respectively. This indicates that the addition of plasticizer DINP may weaken the network structure between the waste LDPE particles and asphalt molecules, thus affecting the high-temperature performance of waste LDPE-modified asphalt. The softening point of modified asphalt with 4% waste LDPE dosing decreased by 2.12%, 3.23%, 6.02%, and 10.25% for 2%, 2.5%, 3%, and 3.5% DINP dosing, respectively. This indicates that the softening point of waste film LDPE/plasticizer DINP-modified asphalt is less affected by the plasticizer DINP when the waste LDPE dosing reaches 4%.

As shown in Figure 5b, the asphalt penetration decreases with the addition of waste LDPE, becoming significantly lower than the penetration of the virgin binder. Upon the addition of the plasticizer DINP, the penetration of the modified asphalt significantly increased. Specifically, when the waste LDPE content was 2%, the modified asphalt with the addition of 2%, 2.5%, 3%, and 3.5% plasticizer DINP showed the most significant increases, rising by 56.8%, 74.8%, 108%, and 115%, respectively, compared with the modified asphalt containing only 2% waste LDPE. However, the growth in penetration began to level off as the proportion of waste LDPE added reached 3%. Among the dosages, the plasticizer DINP dosages of 2.5% and 3% were found to be closest to the penetration of the virgin binder, measuring 66.06/0.1 mm and 69.8/0.1 mm, respectively.

Figure 5c shows that the 5 °C ductility exhibits a decreasing trend with increasing waste LDPE doping, and that it even falls below that of the virgin binder after exceeding 4% waste LDPE doping, indicating that waste LDPE weakens the low-temperature performance of asphalt. Therefore, it is inappropriate to use more than 4% waste LDPE. However, the addition of plasticizer DINP increases the asphalt’s ductility. When the content of waste (LDPE) was 4%, the ductility of all modified asphalts with the addition of the plasticizer DINP increased by 70.7%, 109%, 143%, and 171%, respectively. This may be because the plasticizer DINP is an oily liquid that increases the proportion of lighter components in the asphalt, improving its ductility.

The 25 °C elastic recovery rate refers to the percentage of the recoverable deformation of the asphalt sample when it is stretched to a certain extent. The greater the elastic recovery rate, the better the recovery ability of the asphalt sample after deformation, which indirectly proves that the asphalt has better fatigue resistance. It can be seen from Figure 5d that, when the dosage of waste LDPE is constant or the content of the plasticizer DNIP is constant, the elastic recovery rate of the asphalt increases with increasing content, which indicates that both waste LDPE and DINP can improve the elastic recovery properties of asphalt. However, when the content of waste LDPE exceeds 4%, the growth rate begins to slow. This may be due to there being an excessive content of waste LDPE, which is not easily compatible with asphalt and cannot be fully dispersed inside the asphalt, which thus affects the elastic recovery properties of the asphalt.

In summary, from the perspective of high-temperature performance, the waste LDPE dosage is appropriate at 3% to 5%, but a 5% dosage results in poor low-temperature performance of the modified asphalt. From the perspective of low-temperature performance, a plasticizer DINP dosage above 2.5% is appropriate, but a 3.5% dosage leads to poor high-temperature performance of the modified asphalt. Therefore, the dosage of waste LDPE was 3% and 4%, and the amount of plasticizer DINP was 2.5% and 3% for the follow-up tests.

### 4.3. Rheological Property Analysis

#### 4.3.1. G*/sin *δ*, Phase Angle *δ*, G*sin *δ* and G*cos *δ*

**Figure 6:**
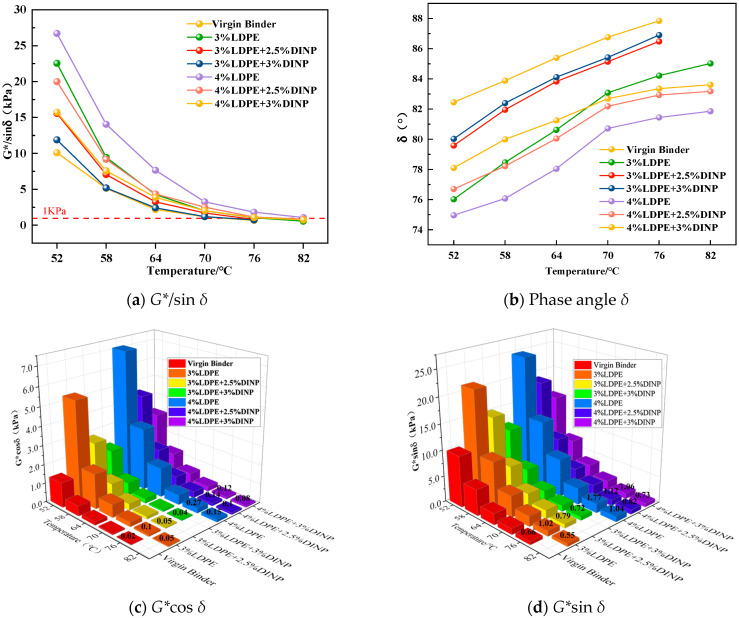
Temperature scanning test results.

Figure 6a shows that, in the temperature range from 52 °C to 70 °C, all the asphalt samples satisfy the specification requirement of G*/sin *δ* being greater than 1 kPa. At 76 °C, only the modified asphalt mixed with 4% waste LDPE and 3% waste LDPE alone met the specification requirements. At 82 °C, only 4% of the waste LDPE-modified asphalt met the specification requirements. In addition, at identical temperatures, the G*/sin *δ* of modified asphalt with different dosages of waste LDPE and plasticizer DINP is 1.8 times greater than that of the virgin binder. However, with increasing amounts of plasticizer DINP, the G*/sin *δ* of the modified asphalt began to decrease, which had an adverse on the G*/sin *δ* of the modified asphalt. However, the G*/sin *δ* of the 4% waste LDPE/2.5% plasticizer DINP-modified asphalt did not have a significant effect and was still more than 80% greater than that of the virgin binder.

Figure 6b shows that the phase angle *δ* of the modified asphalt mixed with waste LDPE and the plasticizer DINP is significantly lower than that of the virgin binder at different temperatures. At the same temperature, the phase angle *δ* decreases with an increasing dosage of waste LDPE and then increases with an increasing dosage of plasticizer DINP. This is because the addition of waste LDPE reduces the viscous component of asphalt and increases its elastic component. The asphalt began to harden, and its deformation resistance improved at high temperatures. The addition of the plasticizer DINP causes the hardened waste LDPE-modified asphalt to soften and the viscous components to increase, thus affecting the high-temperature performance of the asphalt. Therefore, waste LDPE increases the high-temperature rheology properties of asphalt, and plasticizer DINP decreases the high-temperature rheology properties of asphalt.

Figure 6c,d also show that the values of G*cos *δ* and G*sin *δ*, which originally increased with the increase in the amount of LDPE used, started to show a decreasing trend after the addition of the plasticizer DINP. In addition, the test results of the G*cos *δ* and G*sin *δ* values of the virgin binder, 3%LDPE + 2.5%DINP, and 3%LDPE + 3%DINP composite-modified asphalt were not shown when the temperature was at 82 °C. This is due to the fact that, at this temperature, the bitumen is almost completely transformed into a viscous flow state and is unable to retain its shape, and the absolute values of the G*cos *δ* and G*sin *δ* tests become so low that the tests are unable to show their values; however, they are still higher than the G*cos *δ* and G*sin *δ* values of the virgin binder. This shows that 4% dosing of waste LDPE significantly improved the high-temperature rheological properties of asphalt, but the addition of plasticizer DINP will make the elastic component of asphalt lower than the viscous component of asphalt, leading to a state of asphalt softening which affects the high-temperature performance of the asphalt; when the dosage of plasticizer is 3%, the negative impact is the most obvious. According to the analysis of the high-temperature rheological characteristics shown by the above tests, the appropriate dosage should be found to balance the viscosity and elastic components of the asphalt, which can improve its high-temperature performance and cause its low-temperature performance to be less affected. It is recommended that 4% waste LDPE and 2.5% plasticizer DINP be used to modify the virgin binder.

#### 4.3.2. *VTS* Analysis

**Figure 7:**
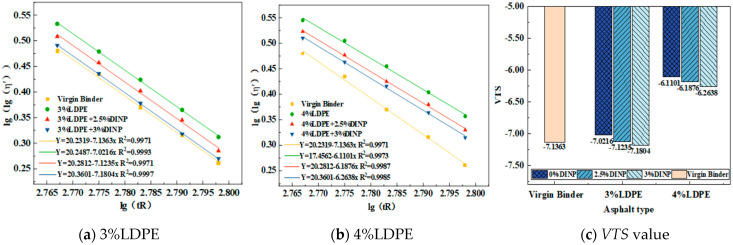
Lg(tR)-lg(lg(*η*’)) fitting curve and *VTS* value.

There exists a certain correlation between the *VTS* index and temperature sensitivity; the smaller the absolute value of the *VTS* index, the better the temperature sensitivity. Using Equation (2), the lg(tR)-lg(lg(*η*′)) fitting curves and *VTS* values of the virgin binder and six kinds of compound-modified asphalt were obtained. As shown in Figure 7a–c, the absolute value of the *VTS* of modified asphalt with 3% and 4% waste LDPE was lower than that of the virgin binder. Among the dosages, the modified asphalt with 4% waste LDPE content had the most significant effect, reducing the VTS value by 14.38% compared to the virgin binder. This demonstrates that waste LDPE can effectively alleviate the influence of the external temperature on the properties of asphalt. After the addition of plasticizer DINP, the absolute value of the *VTS* began to increase with an increasing DINP content. The absolute value of the *VTS* for 3% waste LDPE/3% plasticizer DINP-modified asphalt was even higher than that of the virgin binder. Therefore, the addition of waste LDPE can improve the temperature sensitivity of asphalt, whereas the addition of plasticizer DINP may reduce the temperature sensitivity of asphalt.

#### 4.3.3. Analysis of the Frequency Sweep Test Results

**Figure 8:**
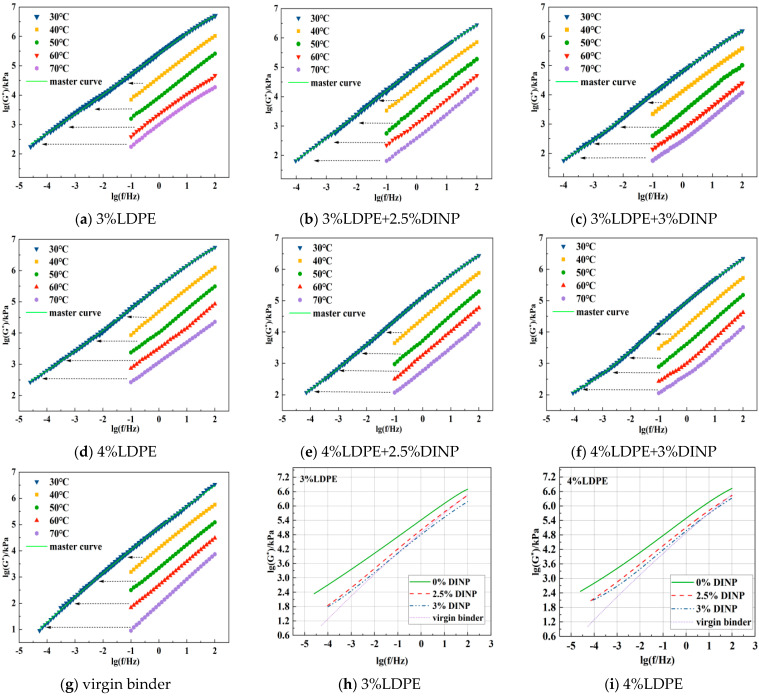
Complex modulus master curves for different asphalts.

Based on frequency scanning results obtained at different temperatures, the complex modulus master curves of different asphalts were obtained based on the time-temperature superposition principle with 30 °C as the reference temperature. As shown in Figure 8a–g, the complex modulus master curves of the different types of asphalt are smooth and continuous with similar shapes. To quantitatively analyze the characteristics of each type of master curve, each type of asphalt under 3% and 4% LDPE was summarized on a single graph, and the master curves of each type of asphalt were fitted according to the sigmoidal model to obtain the fitting parameters, as shown in Figure 8h,i and Table 7.

For the sigmoidal function, *δ* is the minimum value of the sigmoidal function, and the larger its value, the better the high-temperature performance in the low-frequency region. From the fitted parameters in Table 7, it can be seen that the *δ* values of each modified asphalt in the low-frequency region are also larger than those of the virgin binder, and the high-temperature performance is improved. Further, it decreases with the addition of plasticizer. This shows that the plasticizer affects the high-temperature performance of waste LDPE-modified asphalt. It can also be seen that the fitting accuracy of each sigmoidal model to the main curve of the asphalt is close to 1, which indicates high fitting accuracy. From the shape and positional relationship of the main curve shown in Figure 8h,i, it can be seen that, in the low-frequency (high temperature) range, the complex modulus of all waste LDPE/plasticizer DINP-modified asphalt is greater than that of the virgin binder. The complex modulus of asphalt increases with an increasing waste LDPE content, indicating that it has high-temperature deformation resistance and that the 4% waste LDPE-modified asphalt is the best. With increases in plasticizer DINP, the complex modulus in the low-frequency region decreases gradually, but it is still higher than that of the virgin binder. Therefore, the addition of plasticizer DINP has a certain influence on the high-temperature properties of asphalt. For the high-frequency region (low temperature), except for the waste LDPE-modified asphalt without plasticizer DINP, when the plasticizer DINP content exceeds 2.5%, the complex modulus of the modified asphalt begins to be lower than that of the virgin binder, and the low-temperature properties are improved.

#### 4.3.4. BBR Results Analysis

**Figure 9:**
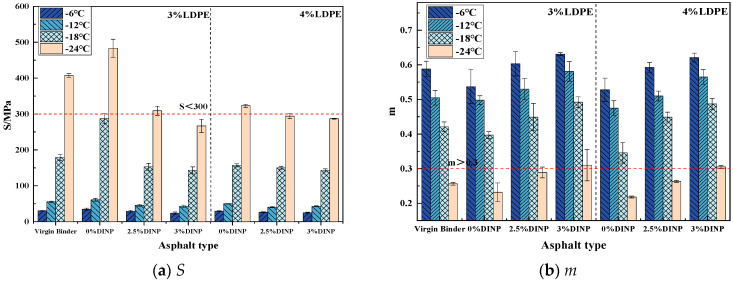
BBR test results.

Figure 9 shows that, at temperatures ranging from −6 °C to −18 °C, all the asphalts meet the specification requirements of the AASHTO standard, with *S* values not exceeding 300 and *m* values greater than 0.3. Among all the asphalt types, only the waste LDPE-modified asphalt without plasticizer DINP exhibited poor low-temperature crack resistance; the stiffness modulus *S* value was greater than that of the virgin binder, and the creep rate *m*-value was lower than that of the virgin binder. The stiffness modulus *S* value of the waste LDPE/plasticizer DINP-modified asphalt with plasticizer DINP was lower than that of the virgin binder, whereas the creep rate *m* was increased and it had excellent low-temperature crack resistance. At −24 °C, only the modified asphalt with 3% plasticizer DINP meets the AASHTO standard [48]. In conclusion, compared with the virgin binder, waste LDPE will affect the low-temperature properties of asphalt, but the addition of plasticizer can significantly improve the low-temperature properties of waste LDPE-modified asphalt. Among the dosages, the improvement effect of 3% plasticizer DINP is the most significant, and this dosage can confer excellent low-temperature cracking resistance on waste LDPE/plasticizer DINP-modified asphalt.

### 4.4. Storage Stability Results Analysis

**Figure 10:**
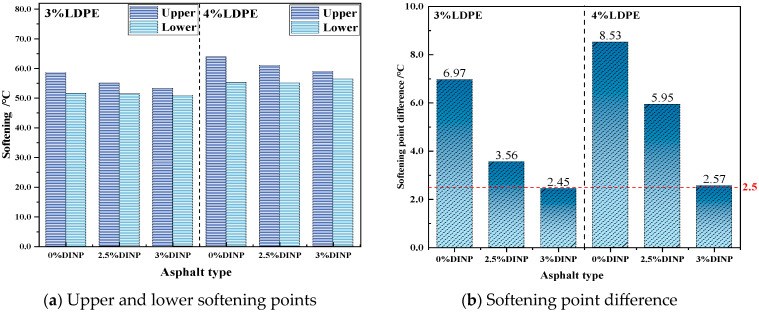
Storage stability of modified asphalts with different contents.

The storage stability of asphalt is a crucial indicator of the changes in the properties of asphalt over long periods of storage. The storage stability of waste LDPE/plasticizer DINP-modified asphalt was evaluated via the softening point difference method in this experiment. Figure 10a,b show that the softening point of the upper part of the modified asphalt is greater than that of the lower portion because the density of the waste LDPE is slightly lower than that of the virgin binder. During high-temperature storage, the polymer begins to separate and migrate upwards to the upper portion of the aluminum tube, approaching the upper softening point. In addition, the softening point difference between 3% LDPE-and 4% LDPE-modified asphalt without plasticizer DINP was significantly greater than the standard value (2.5 °C), and severe segregation occurred in both. However, with an increasing dosage of plasticizer DINP, the difference between the upper and bottom softening points of modified asphalt with the same dosage of waste LDPE decreases gradually. When the dosage of waste LDPE and plasticizer DINP is 3%, the softening point of the modified asphalt is 2.45 °C, which meets the requirements of the relevant specifications. In conclusion, the addition of plasticizer DINP helps to improve the storage stability of waste LDPE-modified asphalt, and the improvement effect of the 3% dosage is the best.

### 4.5. FM Results Analysis

**Figure 11:**
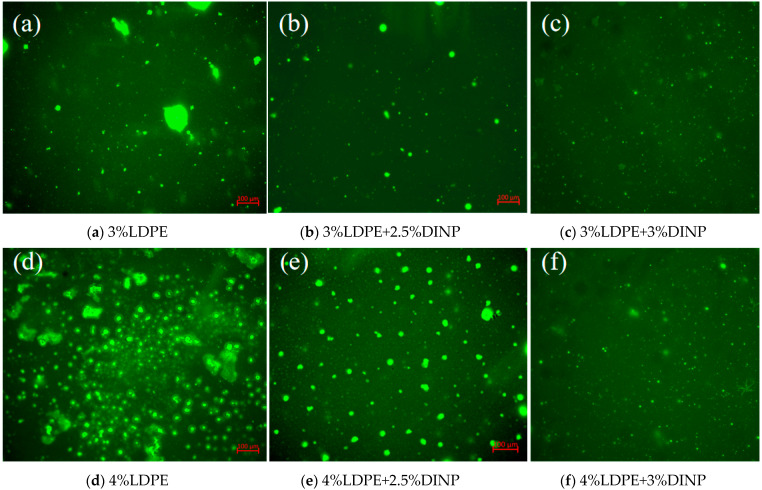
Fluorescence spectra of different modified asphalts.

To analyze the distribution of LDPE in asphalt and evaluate the high-temperature storage stability of waste LDPE/plasticizer DINP-modified asphalt, the microstructures of the six groups of modified asphalt were observed via fluorescence microscopy, as shown in Figure 11a–f.

Figure 11 shows that waste LDPE-modified asphalt without the addition of plasticizer DINP exhibits an agglomeration phenomenon, and that, the higher the waste LDPE dosage, the more serious the agglomeration phenomenon. This indicates that waste LDPE is not sufficiently dissolved in the asphalt, which can easily cause the segregation of asphalt and affect the stability of its high-temperature storage. After adding plasticizer DINP, the particle size of waste LDPE decreases with increases in plasticizer content, and the distribution is uniform, which is consistent with the softening point difference test results. When the content of plasticizer DINP reached 3%, the distribution of the LDPE in the asphalt was the most uniform, with the smallest particles and the best compatibility with asphalt. This indicates that the addition of plasticizer DINP supplemented the light components in the asphalt, balanced its internal structure asphalt, and improved its high-temperature storage stability.

### 4.6. FTIR Results Analysis

**Figure 12:**
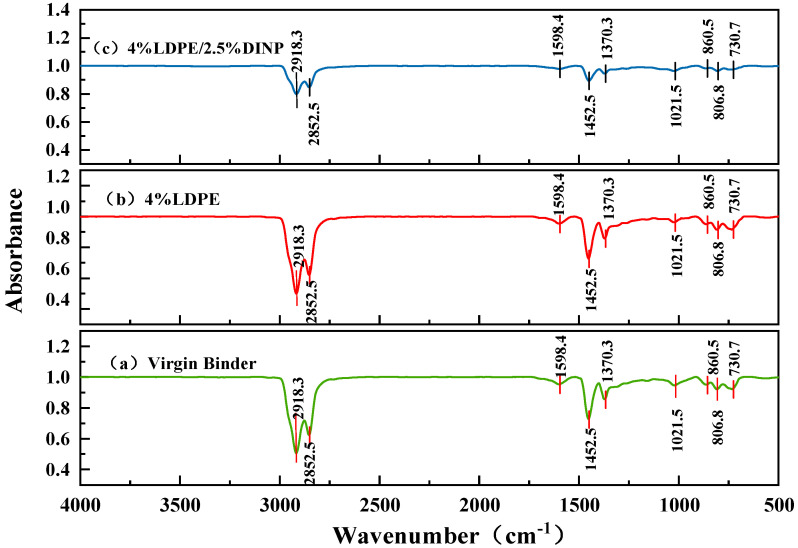
FTIR of asphalts with different surnames.

To investigate the mechanism of the modification of asphalt by waste LDPE and plasticizer DINP, a representative 4% waste LDPE-modified asphalt and a 4% waste LDPE/2.5% plasticizer DINP-modified asphalt were selected and compared with the FTIR spectra of the virgin binder. Figure 12 shows that the FTIR profiles of the virgin binder (a), 4% waste LDPE-modified asphalt (b), and 4% waste LDPE/2.5% plasticizer DINP-modified asphalt (c) are in general agreement. In the functional group region, strong absorption peaks caused by antisymmetric and symmetric stretching vibrations of the methylene-CH_2_ group appeared at 2918.3 and 2852.5 cm^−1^ in the alkane structures. All of the samples presented one weak characteristic absorption peak at 1598.4 cm^−1^, which may be related to the C=C bond stretching vibration in the aromatic group. There are two characteristic absorption peaks at 1452.5 and 1370.3 cm^−1^, generated by in-plane C-H bending vibration and the symmetric angular vibration of methyl-CH_3_, respectively. In alcohol and ester structures, all absorption peaks are at 1021.5 cm^−1^ due to C-O tensile vibration. There are two weak absorption peaks at 860.5 and 806.8 cm^−1^, caused by C-O-C stretching vibration in the aromatic group and appearing in the ether bond structure. There is one characteristic absorption peak at 730.7 cm^−1^, which is generated by the C-H vibration in the substitution region of the benzene ring and is found in aromatic compounds. In summary, no new absorption peak changes occurred in the waste LDPE/plasticizer DINP-modified asphalt, which indicates that there is no chemical reaction between the virgin binder and the waste LDPE or plasticizer DINP, and that the addition of the plasticizer DINP and LDPE does not change the original structure of the virgin binder. the mechanism of the modification of asphalt by these additives is mainly simple physical entanglement, swelling, and adsorption, in addition to other physical modification mechanisms.

## 5. Conclusions

The accumulation of agricultural waste films, especially waste LDPE films, has created various serious environmental problems. This study aims to utilize waste LDPE and plasticizer DINP to modify asphalt binder and thereby improve the properties of asphalt properties and solve the environmental problems caused by waste LDPE films. A series of experiments were conducted to determine the optimal preparation process and blending dosage of waste LDPE/plasticizer DINP-modified asphalt, as well as to evaluate its rheological properties and storage stability. The main conclusions of the study are as follows:

1. Temperature scanning tests and frequency scanning master curve analysis indicated that the waste LDPE/plasticizer DINP-modified asphalt had increasing high-temperature resistance as the dosage of waste LDPE increased. The low-temperature properties of the asphalt improved when the content of plasticizer DINP exceeded 2.5%;
2. The BBR test results indicated that the addition of 3% plasticizer DINP can increase the resistance of waste LDPE/plasticizer DINP-modified asphalt to low-temperature cracking;
3. Storage stability and fluorescence microscopy tests indicated that the addition of 2.5% and 3% plasticizer DINP significantly improved the storage stability of waste LDPE/plasticizer DINP-modified asphalt;
4. When the dosage of waste LDPE was 4% and the dosage of plasticizer DINP was 2.5%, the rheological and storage stability properties of the asphalt binder clearly improved.

Future research directions could be based on the preferred content and preparation methods to further investigate the storage stability and rheological properties of modified asphalt after it is aged to obtain other ideas for its use in green road construction.

## Figures and Tables

**Table 1:** Main technical indicators of waste LDPE.

Density (g/cm^3^)	Melt Flow Rate (g/10 min)	Tensile Strength (MPa)	Softening Point (°C)	Elongation at Break (%)
0.9244	1.83	1.83	95	620

**Table 2:** Shearing process optimization orthogonal experimental design table.

Levels	Factor		
	A Shear Time/min	B Shear Temperature/°C	C Shear Rate/(r/pm)
1	30	170	3500
2	60	180	4500
3	90	190	5500

**Table 3:** Test results of the orthogonal tests.

Test Number	Test Scheme	A	B	C	Softening Point/°C	Penetration (25 °C)/0.1 mm	Ductility (5 °C)/cm
1	A_1_B_1_C_1_	1	1	1	57.7	62.5	6.7
2	A_1_B_2_C_2_	1	2	2	58.8	62.6	9.3
3	A_1_B_3_C_3_	1	3	3	59.2	62.3	12.3
4	A_2_B_2_C_3_	2	2	3	60.2	61.0	8.8
5	A_2_B_3_C_1_	2	3	1	57.8	59.5	6.0
6	A_2_B_1_C_2_	2	1	2	57.5	61.8	13.4
7	A_3_B_3_C_2_	3	3	2	59.0	62.1	7.5
8	A_3_B_1_C_3_	3	1	3	56.2	59.8	7.9
9	A_3_B_2_C_1_	3	2	1	57.9	62.3	6.6

**Table 4:** Effects of the modification process parameters on the softening point.

Indicator	Factor	A	B	C
Softening point/°C	*K*_1_	175.7	171.4	173.4
	*K*_2_	175.5	176.9	175.4
	*K*_3_	173.1	176.0	175.5
	*k*_1_	58.55	57.12	57.80
	*k*_2_	58.51	58.98	58.45
	*k*_3_	57.70	58.66	58.51
	*R*	0.85	1.86	0.71
	Ranking of factors	*R*_B_ > *R*_A_ > *R*_C_		
	Optimal blending parameter	A_1_B_2_C_3_		

**Table 5:** Effects of the modification process parameters on the penetration.

Indicator	Factor	A	B	C
Penetration/0.1 mm	*K*_1_	187.37	184.1	184.3
	*K*_2_	182.31	185.9	186.5
	*K*_3_	184.21	183.9	183.1
	*k*_1_	62.46	61.37	61.44
	*k*_2_	60.77	61.97	62.17
	*k*_3_	61.40	61.29	61.02
	*R*	1.69	0.68	1.15
	Ranking of factors	*R*_A_ > *R*_C_ > *R*_B_		
	Optimal blending parameter	A_1_B_2_C_2_		

**Table 6:** Effects of the modification process parameters on the ductility.

Indicator	Factor	A	B	C
Ductility/cm	*K*_1_	28.30	28.00	19.30
	*K*_2_	28.20	24.70	30.20
	*K*_3_	22.00	25.80	29.00
	*k*_1_	9.43	9.33	6.43
	*k*_2_	9.40	8.23	10.07
	*k*_3_	7.33	8.60	9.67
	*R*	2.10	1.10	3.63
	Ranking of factors	*R*_C_ > *R*_A_ > *R*_B_		
	Optimal blending parameter	A_1_B_1_C_2_		

**Table 7:** Parameters for fitting the master curve of complex modulus of different types of asphalt.

Type of Asphalt	*δ*	*α*	*λ*	*β*	*R* ^2^
virgin binder	7.121	−9.926	0.323	0.431	0.9996
3%LDPE	10.276	−11.386	0.287	0.428	0.9993
3%LDPE + 2.5%DINP	8.165	−9.181	0.349	0.458	0.9995
3%LDPE + 3%DINP	7.901	−7.292	0.397	0.439	0.9998
4%LDPE	12.125	−15.253	0.451	0.529	0.9991
4%LDPE + 2.5%DINP	9.155	−8.772	0.324	0.381	0.9992
4%LDPE + 3%DINP	8.424	−9.403	0.286	0.370	0.9996

## Data Availability

The original contributions presented in this study are included in the article. Further inquiries can be directed to the corresponding author.
